# Supplementary material for: Advancing Mycobacterium leprae transmission research: Insights from the R2STOP fund
Source: PLoS Negl Trop Dis. 2025 Aug 11;19(8):e0013370. doi: 10.1371/journal.pntd.0013370 (PMC12338790; doi:10.1371/journal.pntd.0013370)
Supplement: Attachment — Submitted filename: Palmeirim_R2STOP_Pbp response final_04.07.2025.docx [file pntd.0013370.s002.docx]

**Palmeirim *et al*. “Advancing *Mycobacterium leprae* transmission research: insights from the R2STOP fund”**

**Point-by-point response**

Basel, 04 July 2025

Dear Editor

We refer to your e-mail dated 6^th^ May 2025 and would like to thank you for reviewing our manuscript and for offering useful and constructive comments. In the meantime, we have addressed all the points put forward in the revised manuscript and our point-by-point response is given below. To readily assist you in tracking our changes made, we have also uploaded a version using track-changes.

Marta S Palmeirim (on behalf of all authors)

**Journal Requirements:**

1) Please ensure that the CRediT author contributions listed for every co-author are completed accurately and in full.

At this stage, the following Authors/Authors require contributions: Marta Sólveig Palmeirim, Annemieke Geluk, Bouke C. de Jong, Sofie M. Braet, Kevin R. Macaluso, JoAnn M. Tufariello, Mallika Lavania, Itu Singh, Rahul Sharma, Pushpendra Singh, and Peter Steinmann. Please ensure that the full contributions of each author are acknowledged in the "Add/Edit/Remove Authors" section of our submission form.

The list of CRediT author contributions may be found here: <https://journals.plos.org/plosntds/s/authorship#loc-author-contributions>

Done

2) Please amend your detailed Financial Disclosure statement. This is published with the article. It must therefore be completed in full sentences and contain the exact wording you wish to be published. Please ensure that the funders and grant numbers match between the Financial Disclosure field and the Funding Information tab in your submission form. Note that the funders must be provided in the same order in both places as well.

- State the initials, alongside each funding source, of each author to receive each grant. For example: "This work was supported by the National Institutes of Health (####### to AM; ###### to CJ) and the National Science Foundation (###### to AM)."

None of the authors received funding for writing this manuscript.

- State what role the funders took in the study. If the funders had no role in your study, please state: "The funders had no role in study design, data collection and analysis, decision to publish, or preparation of the manuscript.".

If you did not receive any funding for this study, please simply state: “The authors received no specific funding for this work.”

The funders of the R2STOP funding scheme had no role in study design, data collection and analysis, decision to publish, or preparation of the manuscript.

**Reviewers' Comments:**

**Introduction**

**Reviewer #1:** The introduction and methods sections are clearly written and appropriate. I have no further comments on these parts.

We thank the reviewer for his positive feedback.

**Methods**

**Reviewer #1:** Regarding the results section, I have the following comments and suggestions:

• The amount of funding for the 6 projects varies considerably, ranging from CAD 26,000 to 395,145. However, to interpret the outcomes of each project, it would be important to know how much funding each project received individually. This could be added to Table 1.

We fully understand the reviewer’s point and, as suggested, have added a new column to Table 1 which includes the funding awarded to each project.

• Under the heading ‘Funded project key findings’, the information on each project could be structured more consistently. Now the type and amount of information per project differs considerably, giving the impression of simple copy-paste from other texts. I would suggest 3 standard headings for each description:

1. Title of the project (with name PI)

2. Aim(s) of the study

3. Main findings

With this description I would also suggest that a word count is given to the writers, so that each description is about the same length. In this suggested brief abstract, there is no mention of background, methods, and discussion. The background is usually clear from the title. If necessary, the methodology can be indicated briefly in the aim. (E.g. the aim of our study is to …… by means of ….). Regarding discussion, see my next points.

The section has been rewritten as suggested.

• I think the box with key messages is too diversely formulated for an easy comparison and overview. I like the idea of the boxes and suggest rephrasing it from ‘key messages’ (which I think is vague) to ‘added insight/knowledge’. This is like what journals sometimes ask authors to include in their summary information: what is known and what is the added knowledge generated by this study. The part on ‘what is known’ can be left out here. But the added insight/knowledge is key to the aim of this report and of most interest to the readers. This could be summarized in the boxes with 2-3 bullet points per project.

We have renamed the box and edited its content to provide the relevance for the projects, choosing a text rather than bullet point style.

• After this review of contents and added knowledge, the second part of the results could then focus on operational aspects of the R2STOP funding scheme (PI’s perception). Given the limited amount of meaningful information, I would shorten this part by leaving out Figure 2 and just give a written description without making it sound like a formal questionnaire study (“all respondents …”). I guess the ‘respondents’ are actually the co-authors, so just keep the text straightforward regarding satisfaction on the four aspects mentioned.

Figure 2 has been deleted as suggested, and the paragraph edited.

**Conclusions**

**Reviewer #1:** Regarding the Discussion and Conclusions, I have the following comments:

• I think the discussion is the part where more critical appraisal is possible. Now there are phrases as “we believe that…”, and “the 6 funded projects contributed significantly to our understanding… (says who…!?)”. These and other phrases in the first paragraph of the discussion are just too general and subjective. If the PIs state their results clearly and concretely in textbox 1, it becomes easier to refer to this ‘added knowledge’ and conclude creditably about their contribution.

Subjective statements have been edited to make them authoritative. Also, the section has been edited throughout.

• I also suspect that not all projects went equally well. As a reader, I am (for instance) wondering about the projects of Macaluso and Singh. They both report only 1 publication, while both are 2-year projects. Yet Macaluso has 1 PhD student, and Singh has 8 students (of different levels) involved. So, what went on? It might be explained by differences in budget, or many other things. I would like to know more to understand this better. If there are poor or disappointing results, this could be mentioned (tactfully), because there could be important learning points.

Thank you for drawing attention to the shortcomings. A respective paragraph has now been added to the discussion.

• What are ‘strengths and weaknesses’ in the scheme that could be highlighted? I am sure there are things to be said about budget size, procedure, collaboration, capacity building, risks, etc. What are lessons learnt? Do’s and don’ts, etc.? There is some discussion on this in the second paragraph of the Discussion, but I believe it can be more systematic and elaborate.

This is a very good idea. A respective paragraph has now been added.

• The conclusion introduces several elements that were not really mentioned or reviewed in the previous parts, such as ‘invigorating research’, and ‘establishing solid review infrastructure’. I am sure this is the case, but it was not really addressed and established explicitly in the Results and Discussion sections.

Edits in other parts of the manuscript, especially the introduction and discussion sections, now provide context for these statements.

**Editorial and Data Presentation Modifications**

**Reviewer #1**: The report on page 12 of Sharma and Singh appears to have an incomplete sentence (line 10-12). Also line 6 should read strains and not strain. However, if the authors rearrange these descriptions according to my suggestions, such unclear sentences would hopefully be solved.

We thank the reviewer for drawing our attention to the typo on line 6, which has now been addressed. In line with other comments, the report by Sharma and Singh as been extensively edited.

**Summary and General Comments**

Reviewer #1: In summary, I believe this report can be greatly improved by introducing a more rigorous structure, especially in the results and discussion sections. Being a self-assessment, a more precise report on the projects and a more detailed critical appraisal of the outcomes and impact of the R2STOP fund would benefit information provision and credibility enormously.

Thank you for this critical yet helpful and supportive assessment. We believe that the thorough and extensive revision has substantially improved the credibility and relevance of the manuscript.

**Figure resubmission:**

While revising your submission, please upload your figure files to the Preflight Analysis and Conversion Engine (PACE) digital diagnostic tool, https://pacev2.apexcovantage.com/. PACE helps ensure that figures meet PLOS requirements. To use PACE, you must first register as a user. Registration is free. Then, login and navigate to the UPLOAD tab, where you will find detailed instructions on how to use the tool. If you encounter any issues or have any questions when using PACE, please email PLOS at figures@plos.org. Please note that Supporting Information files do not need this step. If there are other versions of figure files still present in your submission file inventory at resubmission, please replace them with the PACE-processed versions.
